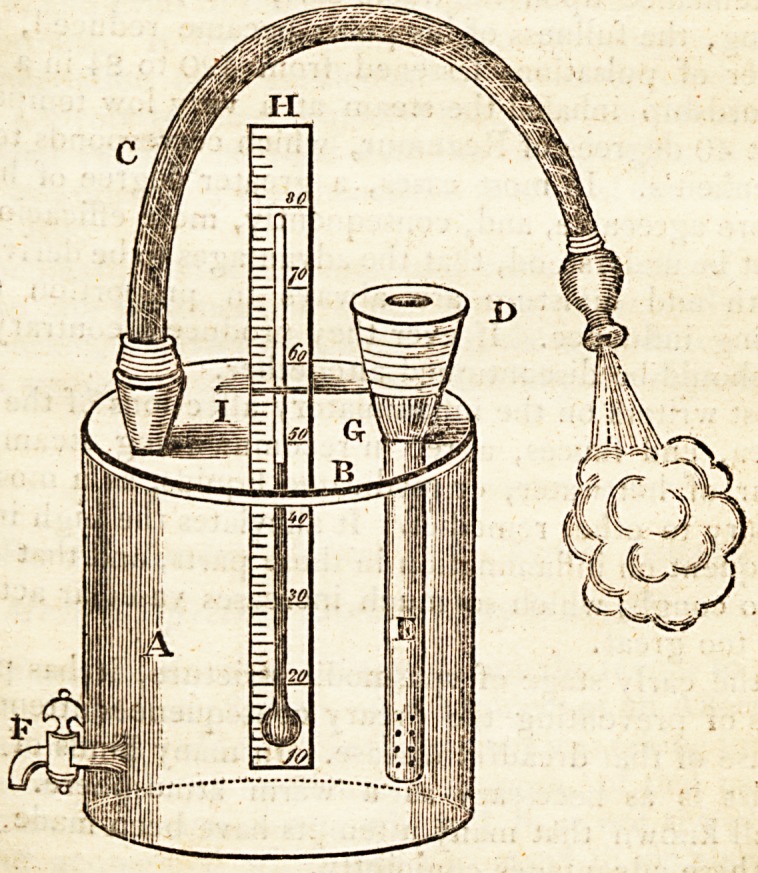# Mr. Machell's Portable Inhaler and Topical Vapour-Bath

**Published:** 1818-02

**Authors:** Thomas Machell

**Affiliations:** Member of the Royal College of Surgeons. 4, *Great Rider-Street, St. James's*


THE LONDON

### Medical and Physical Journal.

2 OF VOL. XXXIX.] FEBRUARY, 1818. [NO. 228.

*To the Editors of the London Medical and Physical Journal.*

GENTLEMEN,

BY giving publicity to an apparatus, approved of by every practitioner who has done me the honour of inspecting it, you will greatly oblige,

Gentlemen,
Your obedient and obliged servant,
THOMAS MACHELL,
Member of the Royal College of Surgeons.

4, *Great Rider-street, St. James's:*
*January* 11, 1818.

*A.*—The receiver, containing about a gallon measure, may be made of metal or earthenware.

*B.*—A lid, or cover, closely fitted to prevent the escape of any steam.

*C.*—A flexible tube of convenient length, one end fixed into the cover *B*, the other to be applied to the mouth for the purpose of inhaling the steam, or to any part of the body, so as to form a topical vapour-bath.

*D* and *E.*—An elastic apparatus on the outside attached to a tube in the inside, (answering all the purposes of a bellows with its nozzle, but much more manageable,) for forcing the vapour from the warm water or medicated fluid contained in the receiver.

*F.*—A stop-cock for drawing off the water, when too hot or too cold for use.

*G.*—The place in which the elastic apparatus is fitted to the tube.

*H.*—A thermometer let into the receiver through the cover at *I*, to regulate the heat of the fluid.

Fresh water, hot or cold, may be added, by unscrewing the elastic apparatus at *G*.

The late Earl Stanhope (for whom the inhaler was invented,) was very sensible, in his last illness, of the benefit he derived from its use. Indeed, I had occasion to observe, in my attendance upon the noble earl, that, immediately after inhaling, the fullness of his pulse became reduced, and the number of pulsations lessened from 120 to 84 in a minute. His lordship inhaled the steam at a very low temperature, viz. at 40 degrees of Reaumur, which corresponds to 122 of Fahrenheit's. In most cases, a greater degree of heat will be more agreeable, and, consequently, more efficacious; for it must be understood, that the advantages to be derived from warmth and moisture are always in proportion to their soothing influence. If ever they produce a contrary effect, they should be discontinued altogether.

Most writers on the inflammatory affections of the thorax, trachea, and fauces, agree in recommending steam, or the vapour of hot water, or medicated liquids, as a most useful auxiliary to other remedies. It alleviates the high irritation consequent on inflammation in those parts, and that disposition to cough, which so much increases vascular action, already too great.

In the early stage of spasmodic stricture, it has proved a means of preventing the dreary consequences attending the increase of that dreadful disease. In many kinds of asthma, a moist is as necessary as a warm atmosphere. This is so well known that many attempts have been made to procure these advantages conjointly.

The late Dr. Mudge obliged the world with an apparatus he invented for the more advantageous exhibition of steam to the fauces, and wrote an ingenious treatise to illustrate the subject. His mechanism, though manageable under his own immediate direction, was attended with insuperable difficulties in other hands. Hence, though the design was excellent, his Inhaler has fallen into disuse.

If, indeed, we reflect upon the insulated situation of the contents of the chest, and that medicines can, in this manner only, be made to come into any thing like contact with the diseased part, and, add to this, the constant sympathy of those parts with the fauces, and the now universally-admitted advantage of a regulated temperature, the necessity of such an apparatus will be as universally admitted in all pulmonary diseases.

In external inflammation, the use of the remedy must be determined, in some measure, by the sensations of the patient, and by the attention of the surgeon. If the patient feels relief, we cannot doubt the efficacy of the remedy, which may be readily accommodated to his feelings.

The above apparatus is contrived in such a manner as to admit of our ascertaining and regulating the degree of thermometrical heat with such certainty and precision, as to preclude the liability of injuring parts of such acknowledged delicacy, and even to render its application safe and easy to other organs. It has been found (on the suggestion and recommendation of a gentleman who has devoted his attention entirely to those subjects) that this method of applying heated vapour, charged with the volatile effluvia of medicinal substances, is very beneficial in several diseases of the eye and ear.

In rheumatic and stiff joints, and various kinds of distortions, to the treatment of which I now particularly devote my attention, I have ascertained that the application of medicated steam or vapour serves materially to expedite the cure.

---

*For the London Medical and Physical Journal.*

## Figures and Tables

**Figure f1:**